# The real-world problem of care coordination: a longitudinal qualitative study with patients living with advanced progressive illness and their unpaid caregivers

**DOI:** 10.1186/1472-6963-14-S2-O27

**Published:** 2014-07-07

**Authors:** Barbara A Daveson, Richard Harding, Cathy Shipman, Bruce L Mason, Eleni Epiphaniou, Irene J Higginson, Clare Ellis-Smith, Lesley Henson, Dan Munday, Veronica Nanton, Jeremy Dale, Kirsty Boyd, Allison Worth, Stephen Barclay, Anne Donaldson, Scott A Murray

**Affiliations:** 1 Cicely Saunders Institute, King’s College London, London, UK; 2 Centre for Population Health Sciences, Medical School, University of Edinburgh, Edinburgh, UK; 3 Health Sciences, University of Warwick, Coventry, UK; 4 The Primary Care Unit, Institute of Public Health, Cambridge, UK

## Objectives

To develop a model of care coordination for patients living with advanced progressive illness and their unpaid caregivers, and to understand their perspective regarding care coordination.

## Design

A prospective longitudinal, multi-perspective qualitative study involving a case-study approach.

## Materials and methods

Serial in-depth interviews were conducted, transcribed verbatim and then analyzed through open and axial coding in order to construct categories for three cases (sites). This was followed by continued thematic analysis to identify underlying conceptual coherence across all cases in order to produce one coherent care coordination model.

## Participants

Fifty-six purposively sampled patients and 27 case-linked unpaid caregivers.

## Settings

Three cases from contrasting primary, secondary and tertiary settings within Britain.

## Results

Coordination is a deliberate cross-cutting action that involves high-quality, caring and well-informed staff, patients and unpaid caregivers who must work in partnership together across health and social care settings. For coordination to occur, it must be adequately resourced with efficient systems and services that communicate. Patients and unpaid caregivers contribute substantially to the coordination of their care, which is sometimes volunteered at a personal cost to them. Coordination is facilitated through flexible and patient-centered care, characterized by accurate and timely information communicated in a way that considers patients’ and caregivers’ needs, preferences, circumstances and abilities.

## Conclusions

In the midst of advanced progressive illness, coordination is a shared and complex intervention involving relational, structural and information components. Our study is one of the first to extensively examine patients’ and caregivers’ views about coordination, thus aiding conceptual fidelity. These findings can be used to help avoid oversimplifying a real-world problem, such as care coordination. Avoiding oversimplification can help with the development, evaluation and implementation of real-world coordination interventions for patients and their unpaid caregivers in the future.

